# Sex Determination in Honeybees: Two Separate Mechanisms Induce and Maintain the Female Pathway

**DOI:** 10.1371/journal.pbio.1000222

**Published:** 2009-10-20

**Authors:** Tanja Gempe, Martin Hasselmann, Morten Schiøtt, Gerd Hause, Marianne Otte, Martin Beye

**Affiliations:** 1 Department of Genetics, Heinrich Heine University Duesseldorf, Duesseldorf, Germany; 2 Department of Population Biology, University of Copenhagen, Copenhagen, Denmark; 3 Biozentrum, Martin-Luther-Universitaet, Halle-Wittenberg, Halle, Germany; Janelia Farm Research Campus, United States of America

## Abstract

Organisms have evolved a bewildering diversity of mechanisms to generate the two sexes. The honeybee (*Apis mellifera*) employs an interesting system in which sex is determined by heterozygosity at a single locus (the Sex Determination Locus) harbouring the *complementary sex determiner* (*csd*) gene. Bees heterozygous at Sex Determination Locus are females, whereas bees homozygous or hemizygous are males. Little is known, however, about the regulation that links sex determination to sexual differentiation. To investigate the control of sexual development in honeybees, we analyzed the functions and the regulatory interactions of genes involved in the sex determination pathway. We show that heterozygous *csd* is only required to induce the female pathway, while the *feminizer* (*fem*) gene maintains this decision throughout development. By RNAi induced knockdown we show that the *fem* gene is essential for entire female development and that the *csd* gene exclusively processes the heterozygous state. *Fem* activity is also required to maintain the female determined pathway throughout development, which we show by mosaic structures in *fem*-repressed intersexuals. We use expression of Fem protein in males to demonstrate that the female maintenance mechanism is controlled by a positive feedback splicing loop in which Fem proteins mediate their own synthesis by directing female *fem* mRNA splicing. The *csd* gene is only necessary to induce this positive feedback loop in early embryogenesis by directing splicing of *fem* mRNAs. Finally, *fem* also controls the splicing of *Am-doublesex* transcripts encoding conserved male- and female-specific transcription factors involved in sexual differentiation. Our findings reveal how the sex determination process is realized in honeybees differing from *Drosophila melanogaster*.

## Abstract 2

Sex determination in honeybees is realized by the *csd* and the *fem* gene that establish and maintain, throughout development, sexual fates via the control of alternative splicing.

## Author Summary

Sexual differentiation is a fundamental process in the animal kingdom, and different species have evolved a bewildering diversity of mechanisms to generate the two sexes in the proper proportions. Sex determination in honeybees (*Apis mellifera*) provides an interesting and unusual system to study, as it is governed by heterozygosity of a single locus harbouring the *complementary sex determiner* gene (*csd*), in contrast to the well-studied sex chromosome system of *Drosophila melanogaster*. We show that the female sex determination pathway is exclusively induced by the *csd* gene in early embryogenesis. Later on and throughout development this inductive signal is maintained via a positive feedback loop of the *feminizer* (*fem*) gene, in which the Fem protein mediates its own synthesis. The findings reveal how the sex determination process in honeybees is realized by the regulation and function of two genes differing from *Drosophila*.

## Introduction

In 1845 Dzierzon reported that the sex in the honeybee (*Apis mellifera*) is determined by the fertilization and non-fertilization of eggs [1], and this was more than 50 years before the discovery of sex chromosomes [2],[3]. Dzierzon's key observation was that a virgin queen that has not taken a mating flight (queens mate only while in free flight away from nest) produces only male progeny. From this result he inferred that unfertilized eggs develop into males, whereas fertilized eggs differentiate into queens and worker bees, which was later confirmed by cytological studies [4]. The unfertilized eggs have a haploid set of 16 chromosomes when compared with fertilized eggs, in which 32 chromosomes were identified [5]. Despite this, neither the fertilization process nor the haploid or diploid state of the eggs provides the sex determination signal in honeybees. This is shown by the regular occurrence of males in inbred crosses that are derived from diploid, fertilized eggs [6]–[9]. This finding led to the hypothesis of complementary sex determination in honeybees, a mechanism that was first provided by genetic studies in another hymenopteran insect, the parasitic wasp *Bracon hebetor* [10],[11]. Fertilized eggs are either homozygous at the Sex Determination Locus (SDL) and differentiate into diploid males or are heterozygous and develop into females. The diploid males, however, don't survive in a bee colony as they are eaten by worker bees shortly after hatching from the egg. Fertile males are produced by the queen's unfertilized, haploid eggs that are hemizygous at SDL.

The single-locus nature of complementary sex determination in honeybees was confirmed by genetic linkage analysis [12],[13], physical mapping [14], and the genetic linkage map [15]. Part of the SDL was characterized by positional cloning and a fine scale mapping approach that led to the identification of the *complementary sex determiner* (*csd*) gene [16]. The gene encodes an SR-type protein and is a potential splicing factor. The *csd* gene satisfies the criteria of a primary signal of complementary sex determination [16]: (1) *csd* exists in at least 15 allelic variants that differ on average in ∼3% of their amino acid residues [17],[18], (2) females are heterozygous and diploid males are homozygous at the *csd* locus [16], and (3) the gene product is necessary for female development [16]. The latter has been shown in RNAi-induced knockdown experiments of *csd*. Females treated with *csd* dsRNA develop entire male gonads, whereas the treatment of males had no sex-transforming effect.

Disappointingly little, however, is known about the regulatory interactions and mechanisms that link sex determination to sexual differentiation. So far we have no evidence that SDL encodes another gene that, in conjunction with *csd*, operates to establish the sex determined state by heterozygosity. We have recently isolated the entire genomic region of SDL and identified the *feminizer* (*fem*) gene, which is the ancestral progenitor gene from which *csd* derived by gene duplication [19] and lies 12 kb upstream of *csd*. The *fem* gene is required for female development as shown by the sexual transformation of the head of *fem*-repressed females. *Fem* activity is not achieved by heterozygosity. Instead, the *fem* pre-mRNAs are sexually processed into the productive female mode. In the current study we characterized the sex-transforming function of other candidate genes located at SDL. We then investigated the function of sex-determining genes in controlling all aspects of development. Our previous studies were restricted to the control of basic aspects of soma differentiation [16],[19], but the signals that specify the sex of germ cells may differ from those utilized in the soma [20]. For example, in *Drosophila* the gene *transformer* (*tra*), which is the likely ortholog of the *fem* gene [19], is required for the sexual development of the soma but not directly for the sexual fate of germ line cells [21]–[23]. We repressed sex-determining genes in early embryogenesis and scored the sexual development of subtle soma and germ line characters. Finally, we analyzed the regulatory interactions of the sex-determining genes and addressed the question of how these interactions are utilized to maintain sexual fate throughout development. In a previous study we proposed that continuously expressed *csd* is a potential source of information to maintain sexual fate throughout development [16]. In order to study these regulatory interactions, we either repressed or expressed genes and assayed the sexual expression of target genes.

Our findings reveal how the regulation and function of the *csd* and the *fem* gene realizes the sex determination process throughout development.

## Results

### Characterization of Genes Present in SDL

**Figure 1:**
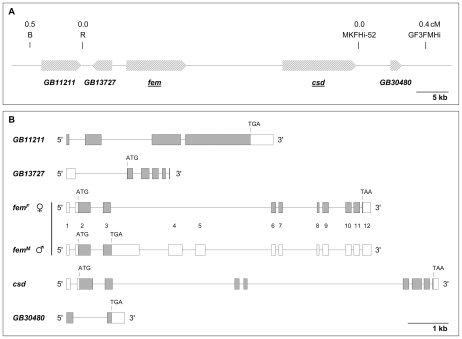
Genomic organization of mRNA producing genes of the SDL. (A) Diagram of genes within the SDL, which is always heterozygous in females as deduced by high resolution genetic mapping [19],[24]. Genes are orientated 5′ to 3′ according to the direction of arrows; the names of functionally characterized genes are underlined. *GB30480* corresponds to gene Ex4.8–5.8 [16]. (B) Exon and intron structure diagram of genes encoded at SDL. Exons are shown as boxes and introns by connecting lines. The deduced open reading frames are marked in grey and the presumed start and stop codons are indicated.

In our previous study we reported the full assembly of the SDL genomic region [19] in a high resolution mapping approach [24]. The involvement of two SDL genes, *fem* and *csd*, in sexual development have thus far been characterized [16],[19]. We hypothesized that this region may harbour additional genes that operate in conjunction with *csd* in the establishment of the primary sex determined state. Three other genes at SDL have been previously predicted, but their involvement in sex determination is unknown. Genes *GB11211* and *GB13727* are located upstream of the *fem* gene, whereas the gene GB30480 (corresponding to Ex4.8–5.8 gene [16]) is located downstream of *csd* (Figure 1). We explored whether the SDL harbours further genes. Potential exons were identified by exon-finding algorithms and homology searches to EST and gene databases. We also identified exons by RT-PCR experiments using cDNA synthesized from embryonic mRNA preparations. Exons testing positive in these experiments were combined, but no further transcription units beside the three previous predicted genes were identified. We extended cDNA fragments using RACE PCR, which resulted in the description of two transcripts (EU101387, EU101392), which corresponds to the two known genes, *GB11211* and *GB13727* (Figure 1A and 1B). The same sequences were isolated from both males and females implying that the transcripts are not sex-specifically processed. We obtained 3,330 bases of the transcript of gene *GB11211*, which divides into four exons (Figure 1B). The 5′ end of this transcript has not been isolated by RACE PCR; thus additional translational start codons may lie upstream from the known transcribed sequence. This partial transcript encodes a 926 amino acid protein with partial similarity to a domain from a *Tribolium castaneum* hypothetical protein (LOC655741) of unknown function. We isolated 803 bases of the transcript of gene *GB13727*, which splits into six exons (Figure 1B) and which is located on the opposite strand from the other SDL genes. The 3′ end of the transcript has not been identified by our RACE experiments. The partial ORF encodes a protein of 193 amino acids. The protein contains a DUF2464 domain of unknown function that is conserved from worms to humans. No other mRNAs have been detected, suggesting that SDL harbours a total of five protein encoding genes. We studied the involvement of genes *GB11211*, *GB13727*, and *GB30480* in sex determination. We injected dsRNA into male and female syncytial embryos in order to repress transcripts of the new genes and recorded gonad differentiation of 5^th^ instar larva. We used gonad differentiation as an informative indicator of sex determination as it is induced early in development [16]. We also analyzed the *fem* gene, for which we have no information on gonad differentiation, and *csd*, which served as a control for entirely switched gonad development. The syncytial female and haploid male embryos were obtained from single-male (drone) inseminated queens and virgin queens, respectively.

**Figure 2:**
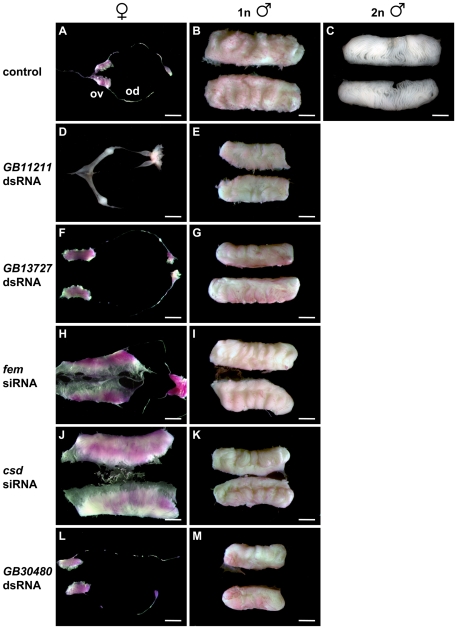
Reproductive organ development of 5^th^ instar male and female larvae in the repression analysis of SDL genes. (A–C) Reproductive organ development of untreated individuals: (A) A pair of normally developed ovaries (ov) and oviducts (od) from an untreated female. (B) A pair of normally differentiated testes from untreated haploid males consisting of densely packed layers of folded testioles. The paired spermducts are not shown. (C) A pair of normally differentiated testes from untreated diploid males consisting of less densely packed layers of folded testioles. The paired spermducts are not shown. (D–G) Repression analysis of gene *GB11211* and *GB13727*. Normally developed gonads of females and haploid males injected with dsRNA devoted to repress the function of gene *GB11211* (D–E) and *GB13727* (F–G). (H–I) Repression analysis of the *fem* gene. (H) Pair of underdeveloped testes from a female treated with *fem* siRNA. The testes of this female individual are covered with oversized epithelial sheaths. The testioles are reduced in length and number when compared with the haploid (B) or diploid (C) males or the pseudomales after *csd* siRNA injection (J). The shape and course of spermducts appear normal. (I) Normally developed testes from a haploid male injected with *fem* siRNAs. (J–K) Repression analysis of the *csd* gene. (J) Pair of fully developed testes from a female treated with *csd* siRNAs. The number, length, and arrangement of testioles resemble entirely of those dissected from diploid males (C). (K) Normally developed testes from a haploid male injected with *csd* siRNA. (L–M) Repression analysis of the *GB30480* gene. Normally developed gonads of females (L) and haploid males (M) injected with *GB30480* dsRNA. Gonads were stained with aceto-orcein (reddish colouring of gonads), which facilitated the dissection process. Scale bars, 1 mm.

Our injections of dsRNAs targeted at repressing the function of the *GB11211* and *GB13727* genes produced individuals with unchanged gonad development (Table 1, Figure 2D–2G), suggesting that neither gene is required for sex determination. We confirmed the knockdown of these genes by showing a reduction of the amount of mRNAs in real time RT-PCR experiments (*t*-test, *p*<0.02 for *GB11211* dsRNAs and *p*<0.001 for *GB13727* dsRNAs treated embryos when compared with mock dsRNA treated controls). Our series of *fem* repression experiments induced by *fem* siRNAs produced 74% females whose gonads had entirely differentiated into male testes (Table 1, Figure 2H). No sex-transformed effects occurred in *fem*-repressed males. The testes of *fem*-repressed females were smaller and contained fewer and shorter testioles in all cases, irrespective of whether we compared larvae of the same age or the same stage with the controls, suggesting that this difference is not just a slowdown of development caused by the effect of RNAi. Nevertheless, this finding extends our previous observations [19] and suggests that *fem* gene products are also required for female gonad differentiation. Our series of *csd* knockdown experiments induced by siRNAs resulted in females with fully developed, normal-sized male gonads (Table 1, Figure 2J). Males treated with *csd* siRNAs showed unchanged development. Knockdown of *GB30480*, the most downstream gene of the SDL locus, did not influence the differentiation of male or female gonads (Table 1, Figure 2L, 2M). Taken together, our knockdown experiments indicate that no mRNA-encoding genes at SDL other than *fem* and *csd* have sex determination functions.

**Table 1:** Gonad development of 5^th^ instar larvae treated with dsRNAs and siRNAs.

Treatment	Females				Haploid Males			
	Number of Embryos	Number of Recovered	Number of Sexual Transformed	Relative Transformation (%)	Number of Embryos	Number of Recovered	Number of Sexual Transformed	Relative Transformation (%)
Non-treated	246	55	0	0	44	7	0	0
mock dsRNA	87	17	0	0	n.p.	n.p.	n.p.	n.p.
Mock siRNA	193	67	0	0	509	33	0	0
*GB11211* dsRNA	135	52	0	0	137	10	0	0
*GB13727* dsRNA	226	38	0	0	126	9	0	0
*fem* siRNA	545	159	118	74	900	48	0	0
*csd* siRNA	520	187	164	88	542	41	0	0
*GB30480* dsRNA	206	31	0	0	121	12	0	0

n.p., not performed.

### The Role of *fem* and *csd* Gene in Soma and Germ Line Differentiation

In *Drosophila* the *tra* gene—the proposed functional and structural ortholog of the *fem* gene [19]—does not dictate the sexual fate of female germ cells, but controls all aspects of somatic differentiation [20],[21]. Combined with our results of smaller testis formation in *fem*-repressed individuals, we hypothesized that the *fem* gene of the honeybee is also not involved in the sexual differentiation of germ cells. To dissect the involvement of *fem* and *csd* in the sexual fate of either the germ or the soma, we injected *fem* or *csd* siRNAs into syncytial embryos, but in this study we reared individuals to the late pupal stage (P3). In these experiments we injected into embryos that were derived from two inbred and three non-inbred crosses. The inbred crosses naturally produce 50% diploid male and 50% female progeny, which we identified by genotyping *csd* alleles. This allowed us to compare the male-transformed characters of diploid females with wild type characters of diploid males. This is of importance as characters of haploid and diploid males can differ slightly.

**Figure 3:**
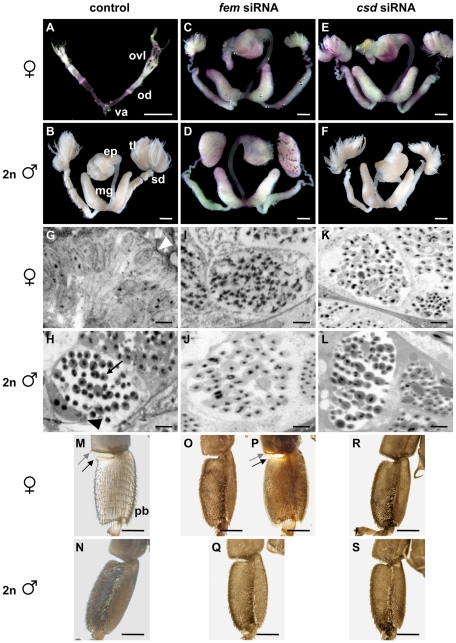
Soma and germ line development of female and diploid male late pupae in the knockdown analysis of the *fem* and the *csd* gene. (A–F) Development of the reproductive organ. (A) Normal pair of ovaries, oviducts (od), and unpaired vagina (va) of an untreated female (worker bee). The ovaries are composed of less than five ovarioles (ovl). (B) Normally developed pairs of testes, spermducts (sd), mucus glands (mg), and unpaired endophallus (ep) of a diploid male. The testes consist of hundreds of thickly packed and folded testioles (tl). (C) Male reproductive organ from a female injected with *fem* siRNAs. The testes are reduced in size and composed of fewer testioles of reduced length. (E) Male reproductive organ from a female treated with *csd* siRNAs. The testes from these pseudomales are of normal size and structure and appear equivalent to the testes from diploid males (B). (D and F) Normally differentiated reproductive organ from a male treated with *fem* or *csd* siRNAs, respectively. Reproductive apparatus was stained with aceto-orcein (reddish colouring), which facilitated the dissection process. Scale bars, 1 mm. (G–L) Differentiation of germ cells in microscopic sections through ovarioles and testicular tubules. (G) Undifferentiated cells in an ovariole of an untreated female. The ovariole is surrounded by an epithelial sheath (white arrowhead). (H) Bundles of spermatids in a testicular tubule (testioles) of a non-injected diploid male. The testicular tubules are composed of spermatocystes containing the spermatids (black arrow) and nurse cells (black arrowhead). (I) Spermatids formed in a fully male differentiated testis of a *fem* siRNA injected female. (K) Spermatids in a fully male-like developed testis from a *csd* siRNA treated female. (J and L) Normal testis and germ cell differentiation of *fem* (J) and *csd* (L) siRNA injected diploid males. Sections were stained with toluidine blue. Scale bars, 10 µm. (M–S) Development of the inner tibia and tarsus surface of the left hind leg. (M) Normally differentiated pollen brush, pollen comb, and lobe of an untreated female worker. The first tarsal segment displays symmetrically arranged rows of bristles, which are used to brush pollen from the body surface (pollen brush, pb). The upper posterior part of the first tarsal segment forms a lobe (black arrow). Spines at the distal part of the tibia form the pollen comb (grey arrow) in which pollen is detached from the pollen brush. (N) Normally developed tibia and first tarsal segment of non-injected diploid males that lack the symmetrical organization of bristles (pollen brush), the lobe, and the pollen comb. (O) Male differentiated hind leg from a female injected with *fem* siRNAs. (P) Development of a mosaic intersex upon *fem* siRNA injections. The posterior part of the first tarsus segment is male, lacks the female-specific lobe (black arrow), and displays bristles in a non-arranged pattern. The anterior part of the tarsal segment is female and shows the symmetrical arrays of bristles. The distal part of the tibia harbours the spines of the pollen comb (grey arrow) indicating a fully developed female structure. (R) Male developed hind leg from a female treated with *csd* siRNAs. (Q and S) Normally developed hind legs from *fem* (Q) and *csd* (S) siRNA injected diploid males. Scale bars, 1 mm.

Repression of the *fem* gene resulted in 74% and 85% of the females (derived from the non-inbred and inbred crosses, respectively) showing all aspects of male differentiation (Table 2). These pseudomales have fully developed internal male reproductive organs, including pairs of testes, mucus glands, and an endophallus (Figure 3C). The pair of testes is, however, reduced in size when compared with those of diploid males. Upon microscopic analysis of sections of testicular tubules we observed elongated bundles, the spermatids, but no indication of the undifferentiated cell type that we found in sections of ovariole tissue from untreated females (Figure 3I). In some sections we found empty testicular tubules (unpublished data) implying that some mature spermatids had already migrated into the seminal vesicles. The fully switched germ cells indicate that *fem* controls germ cell differentiation as well as the soma. The tibia and the first tarsus of sexually transformed female hind legs have a male-like shape (Fig 3O) and lack the female-specific structures such as the pollen basket (unpublished), the pollen comb, and the pollen brush, which is composed of symmetrically arranged rows of bristles (see the hind leg of an untreated female Figure 3M for a comparison). In 7% of *fem* siRNA treated females derived from non-inbred cross (Table 2) we found both disordered (male-like) and symmetrical (female-like) bristles adjacent to one another on the first tarsal segment (Figure 3P). In addition, the individual in Figure 3P lacks the female-specific lobe on the first tarsus but displays the pollen comb on the tibia. This suggests that these hind legs are composed of fully differentiated male and female structures. Knockdown of *csd* produced 76% females that displayed all the aspects of male differentiation in external and internal morphology (Table 2, Figure 3). These pseudomales had fully developed male reproductive organs (Figure 3E) and, in contrast to the *fem*-repressed individuals, testes of normal size. The hind legs showed the full spectrum of male structures (Figure 3R). Upon examination of the cytology of testicular tubules we observed the structures of spermatids in all cases (Figure 3K). The repression of *fem* or *csd* in diploid males (Table 2, Figure 3) does not affect the internal and external morphology, indicating that neither *fem* nor *csd* activity is necessary for male differentiation. These experiments imply that the paralogous gene pair *fem* and *csd* are required for controlling female differentiation of both the soma and the germ cells.

**Table 2:** Sexual development of late pupae (P3) treated with siRNAs.

Treatment	Number of Crosses	Number of Embryos	Females					Diploid Males		
			Number of Recovered	Number of Sexual Transformed	Relative Transformation (%)	Number of Gynander	Relative Gynander (%)	Number of Recovered	Number of Sexual Transformed	Relative Transformation (%)
Non-treated	4 non-inbred	197	44	0	0	0	0	—	—	—
	2 inbred	390	39	0	0	0	0	13	0	0
*fem* siRNA	3 non-inbred	336	27	20	74	2	7	—	—	—
	2 inbred	223	20	17	85	0	0	24	0	0
*csd* siRNA	2 inbred	204	29	22	76	0	0	19	0	0

### Identifying the Regulatory Relationships between *csd*, *fem*, and the *Am-dsx* Gene

Our analyses have identified *csd* and *fem* as different components of the sex determination pathway that are required for all aspects of female differentiation. The next obvious component of the pathway is the *doublesex* (*dsx*) gene. *Dsx* is a transcription factor that in *Drosophila* controls the activity of the final target genes necessary for both male and female somatic differentiation [25],[26]. The sex-specific activity of the *Drosophila* gene is brought about by sexually processed transcripts encoding polypeptides that have male- and female-specific domains at their carboxyl-termini. The female splice pattern is mediated by the female Tra protein [27],[28], which is the proposed ortholog of the Fem protein [19]. Previous studies have identified the ortholog, *Am-dsx*, in the honeybee genome [29]–[31] encoding an atypical zinc-finger domain, the so called OD1 domain. The gene expresses sex-specific mRNAs and presumably proteins. The central role of *dsx* orthologs in sexual differentiation of insects has been provided by the housefly *Musca domestica* [32] and in the moth *Bombyx mori* [33], but functional evidence for the honeybee are so far missing. In order to determine the regulatory interactions within the honeybee sex determination pathway, we generated pseudomales by the injection of *csd* or *fem* siRNAs and examined the sexual expression of *fem* and *Am-dsx* mRNAs in 5^th^ instar larvae by RT-PCR targeting fragments that corresponds to female- and male-specific mRNAs. If the activity of the gene is repressed in females, we expect to find the male mRNAs of downstream components.

**Figure 4:**
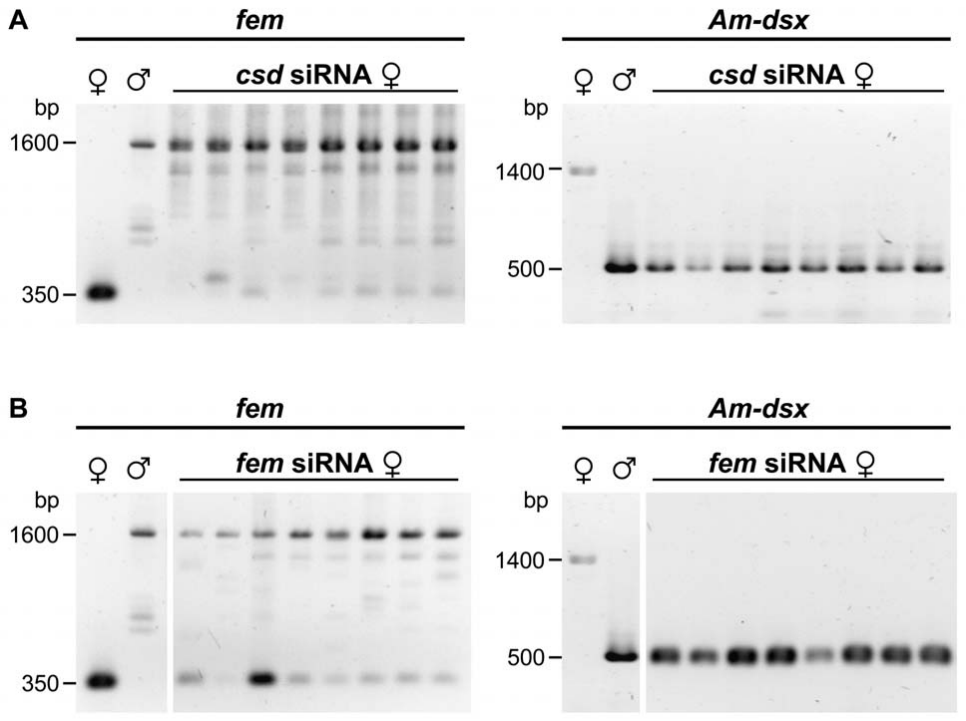
The processing of *fem* and *Am-dsx* transcripts in the response to the knockdowns of the *csd* and the *fem* gene induced by RNAi. (A) The male and female *fem* and *Am-dsx* mRNAs of eight 5^th^ larval instar pseudomales that have been injected with *csd* siRNAs. Fragments corresponding to the *fem* female (∼350 bp) and male (∼1.6 kb) mRNAs and the *Am-dsx* female (∼1.4 kb) and male (∼500 bp) mRNAs were amplified by RT-PCR and resolved by agarose gel electrophoresis. The fragments obtained from untreated females and males are shown in the 1^st^ and 2^nd^ lane, respectively. (B) Same analysis as in (A) except that eight pseudomales have been treated with *fem* instead of *csd* siRNAs.

Pseudomales produced through the repression of *csd* predominantly displayed fragments that correspond to male *fem* and *Am-dsx* mRNAs (Figure 4A), implying that the production of female *fem* and *Am-dsx* mRNAs require *csd* activity. This finding is consistent with the expectation that *csd* is the primary signal that determines all aspects of female differentiation. *Csd* activity is, however, not required to induce male *fem* or *Am-dsx* mRNAs, indicating that the male transcripts do not require any sex-specifying signal and that this is the default regulatory state. The repression of *fem* also produced pseudomales that have male *fem* and *Am-dsx* mRNAs (Figure 4B). The finding of male *Am-dsx* mRNAs in *fem*-repressed females indicates that *fem* activity is necessary to induce female *Am-dsx* mRNAs. The observation of male *fem* mRNAs also suggests that *fem* mRNA production was resumed in later developmental stages after *fem* activity was experimentally repressed in early embryos. From the presence of male mRNAs under conditions of resumed *fem* mRNA production, we conclude that the *csd* gene has lost its ability to direct the processing of *fem* into the female mode. Thus, it appears that in the absence of the female-specifying signal, the male variant is produced that is the default regulatory state.

### Maintenance of the Sex Determined State

**Figure 5:**
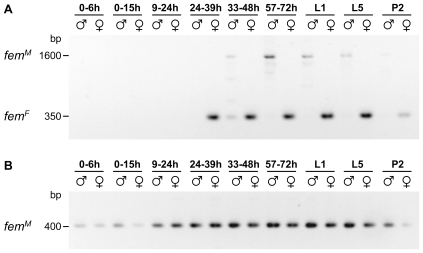
Developmental profile of *fem* mRNA expression. Fragments corresponding to female (A) and male (B) *fem* mRNAs were independently amplified by RT-PCR and resolved by agarose gel electrophoresis. The weak ∼1,600 bp fragments observed in reactions devoted to amplify the female-specific fragment correspond to the male mRNAs. Differences in the amount of cDNAs in the different samples were adjusted prior to PCR amplifications. For the embryonic stages the hours after egg deposition are indicated. The early blastoderm is formed ∼12 h after egg deposition. L1 and L5 are 1^st^ and 5^th^ instar larvae, P2 are pupae at medium stage.

**Figure 6:**
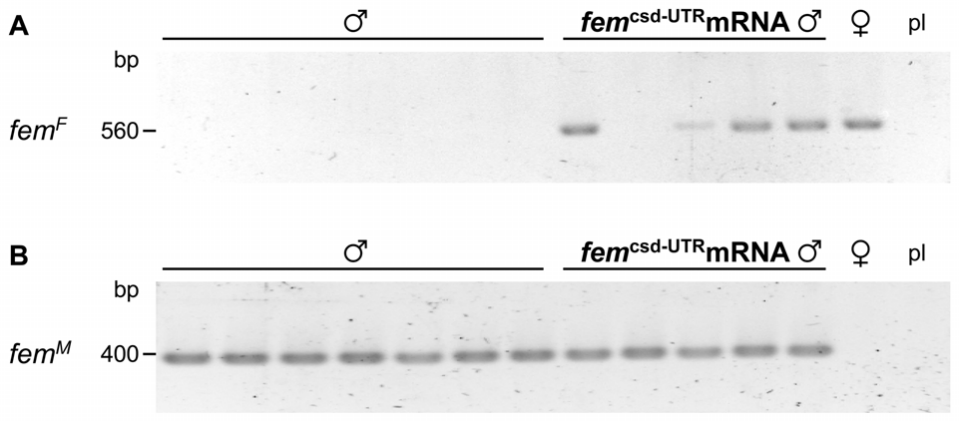
The processing of endogenous *fem* transcripts in response to the injection of Fem encoding mRNA in haploid males. (A) Fragments corresponding to the female *fem* mRNAs of individual 72-h-old embryos were amplified by RT-PCR and resolved by agarose gel electrophoresis. The identity of the female fragments was confirmed by nucleotide sequence analysis. The last lane shows the reactions in which the (pl) *fem* ^csd-UTR^ mRNA encoding plasmid (pfem^csd-UTR^) was used as a template. The absence of a fragment in this high copy DNA control strongly suggests that our primer oligonucleotides will not amplify fragments corresponding to the injected Fem encoding mRNAs (*fem* ^csd-UTR^ mRNA). (B) Amplified fragments corresponding to the male *fem* mRNAs on the same set of samples as described in (A).

Our data so far indicate that the ability of *csd* to direct the processing of *fem* is restricted to a critical window in early development. This prompted us to investigate how the sexual state induced by the *csd* gene is maintained throughout development. Examination of the expression of *fem* during development indicates that female *fem* mRNAs are present in the late blastoderm stage and remain expressed throughout embryonic, late larval, and pupal development (Figure 5). Together with our finding that *csd* is not employed to direct the processing of *fem* in late larvae and pupae, we conclude that an additional mechanism of regulation exists to maintain *fem* processing into the productive female mode in these late stages. In *Drosophila*, the sex determination gene *Sex-lethal* (*Sxl*) encodes a protein that directs the splicing of its own transcript into the productive female mode [34],[35]. This self-splicing loop maintains the female-determined state throughout development [36]. A similar mechanism has been proposed for the medfly *Ceratitis capitata* at the level of the sex-determining *tra* (*Cc-tra*) gene [37],[38], a gene which has a common ancestry with the *fem* gene [19]. These findings prompted us to hypothesize that a positive autoregulatory activity of *fem* provides the mechanism for maintaining the female-determined state and provides a source of a female-specific signal implementing the sexual development pathway. To test our hypothesis we transiently expressed the *fem* gene in males by injecting *fem* encoding mRNAs that we synthesized in vitro. We assayed the processing of the putative target—the endogenous *fem* mRNA—by RT-PCR amplifications. This experiment shows (Figure 6) that expression of *fem* induces a partial switch from male into female mRNAs, suggesting that the provided *fem* activity trans-activates the endogenous *fem* gene. We conclude from this finding that expression of Fem protein induces its own synthesis by directing the processing of *fem* pre-mRNA into the productive female mode. This finding would establish a positive regulatory feedback loop at the level of the *fem* gene.

## Discussion

### The Foundation of Complementary Sex Determination Is a Single Gene, *csd*

Sex in the honeybee is determined by the heterozygosity and hemi/homozygosity at a single SDL. In this study we show that this genomic locus harbours five mRNA encoding genes but that only the *csd* gene fulfils criteria of a primary signal of complementary sex determination, implying that the molecular nature of complementary sex determination is *csd*. When *csd* is repressed in early embryogenesis in females, we see an entire switch into male development that affects all visible aspects of soma and germ line development. We see comparable effects in the soma and the germ line when we repress *fem* in females, which is located 12 kb upstream of *csd*. *Fem* mRNA is, however, sex-specifically processed in response to the heterozygous *csd* gene, suggesting that *fem* is a target that directly responds to the activity at the *csd* gene. We have not identified allelic variants of *fem* transcripts that could encode different allelic specificities within our crosses (unpublished data). We conclude that *fem* is the target of *csd* that implements male or female differentiation by a productive female, or a non-productive male splice variant. The three other mRNA encoding genes of SDL are not necessary for sex determination. Our experiments do not exclude the possibility that SDL harbours a non-mRNA encoding gene (such as a microRNA), but we find no evidence for such a gene. The exclusion of other sex-determining factors at SDL suggests that complementary sex determination entirely relies on heterozygous combination of the *csd* gene. The primary signal of the honeybee is a switch gene that has two regulatory states, the active female and the non-active male state. We propose that induction of the female pathway through gain of *csd* activity is due to the presence of two Csd proteins derived from different alleles. Lack of *csd* activity, and thus male development, results when Csd proteins are derived from the same allele. We hypothesize that activation in females relies entirely on the binding differences of Csd's RS- and asparagine/tyrosine-enriched domains. This region harbours elevated nucleotide polymorphism and has been proposed as the allele specifying domain in our population genetics analysis [17],[18].

The heterozygous activation of *csd* has some analogy to self-incompatibility systems in plants and fungi [39]–[41], in which different alleles of a single locus initiate a developmental program, that in the case the S-locus system in plants controls the outgrowth of a pollen grain. Molecular studies in these systems revealed that the determination of heterozygous state relies on the operation of two separate but closely linked genes [39]–[41]. Our analysis indicates that the heterozygous state in the honeybee is processed by a single gene. The comparison suggests a novel mechanism of gene regulation to those previously identified.

### The *fem* Gene Implements and Maintains Soma and Germ Line Development

In order to determine sex, the activity of heterozygous *csd* must implement sexually different activities to downstream genes. Our identification of the *fem* gene also allowed us to identify female-specific mRNAs [19]. Our analysis shows that the female *fem* mRNAs are required for all visible aspects of female morphology. Testes were, however, all smaller in *fem*-repressed females when compared with wild type diploid males or *csd*-repressed females. We speculate that the timing of *fem* and *csd* activity may affect differently gonad differentiation when we experimentally repress these genes. These testes form normally differentiated testicular tubules harbouring fully developed spermatids, implying that the *fem* gene also controls germ cell differentiation. Despite a common evolutionary origin, the *tra* gene of *Drosophila* does not dictate the sexual fate of germ cells and its function is restricted to the control of the soma [21]–[23].

**Figure 7:**
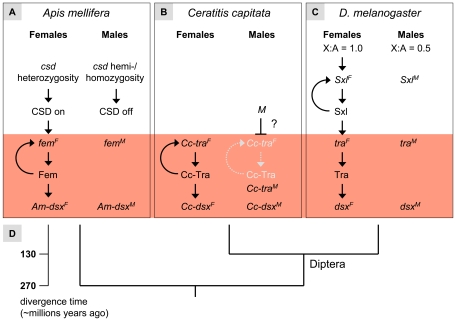
The regulative hierarchy of honeybee sex determination in relation to other insect model species. (A) Model for the honeybee sex determination pathway that controls both soma and germ cells. The heterozygous or homo-/hemizygous state of the *csd* gene determines whether Csd protein is active. Active Csd proteins, derived from different *csd* alleles in females, are splicing factors that direct the processing into female *fem* mRNAs. Female *fem* mRNAs (*fem^F^*) are producing active Fem proteins that are required to mediate the splicing of *Am-dsx* pre-mRNA into the female mRNAs. The Fem protein has an additional positive feedback activity that directs the processing of *fem^F^* mRNAs. Inactive CSD proteins, when derived from homo- or hemizygous *csd* alleles, result in a splicing of the *fem* and *dsx* transcripts, which is the default male state (*fem^M^*, *Am-dsx^M^*). (B) Model for the sex determination pathway in *Ceratitis capitata* [37],[38]. The presence or absence of an unidentified factor *M* determines sex. In the absence of *M* the maternal provided *Cc-tra* gene product establishes an autoregulative loop in which Cc-Tra protein mediates the production of female *Cc-tra* mRNA. The Cc-Tra protein directs the splicing of *Cc-dsx* pre-mRNA into the female mode. The presence of *M* impairs the positive autoregulative loop of the *Cc-tra* gene products producing a default splicing pattern of *Cc-tra* transcripts, the male pre-mRNA. The male *Cc-dsx* mRNA is produced by default. (C) Simplified view of the somatic sex determination hierarchy in *D. melanogaster* [22]. The X∶A ratio determines whether *Sxl* is activated. Sxl protein in females is a splicing factor that directs the splicing of *tra* pre-mRNA into the female mode, resulting in the production of active Tra protein in females. Tra protein mediates the processing of female *dsx* mRNAs. In the absence of Sxl protein all these regulatory decisions do not occur and the male *dsx^M^* is produced by default. The male and female *dsx* transcripts encode sex-specific transcription factors that have several target genes and are involved in various aspects of sexual differentiation. (D) The evolutionary relationship of the species used in the comparison with their approximate time scale of divergence.

We have used the sex-specific mRNAs as a phenotype to identify the interactions of sex-regulatory genes by assuming that genes affecting *fem* and *Am-dsx* splice patterns are operating upstream in the cascade. The *Am-dsx* gene, which is an ortholog of the *dsx* gene of *Drosophila*, is another component of the sex determination whose mRNA is sex-specifically processed [29],[31]. Our analysis indicates that interactions occur at least at three levels of the cascade—*csd* controls splicing of *fem* pre-mRNA, and *fem* regulates the processing of *Am-dsx* pre-mRNAs (Figure 7A). The observation of the male variant of *fem* and *Am-dsx* mRNAs in the repression experiments implies that the female pathway is actively regulated and Csd protein directs splicing in *fem*, and Fem protein directs splicing in *Am-dsx* female mRNAs. The male pathway is the default regulatory state that does not require any sex-specifying control. This suggests that the male splice pattern of *fem* and *Am-dsx* results from the splice machinery that is present in both males and females. Our analysis does not preclude other levels of interactions. It is conceivable that the Fem protein is directly involved in the splicing process of *Am-dsx* transcripts given the apparent structural and functional relationship of these genes to the *Drosophila tra* and *dsx* genes [19],[22],[29],[31]. To show a direct interaction between these levels of regulation requires detailed protein binding and splicing studies, however. Besides the succession of interactions, we find evidence for a positive regulatory feedback loop at the level of the *fem* gene (Figure 7A). We have demonstrated that provisioning of female *fem* mRNAs in males induces a partial shift in the processing of endogenous *fem* pre-mRNAs into the female mode. We conclude from this observation that the expression of *fem* establishes a positive feedback loop in which Fem protein induces—directly or indirectly—its own synthesis by splicing *fem* pre-mRNAs into the productive female mode. In males, *fem* activity would be absent and the pre-mRNA would be spliced into the non-reproductive male mode. We propose that the positive feedback loop would (1) generate stably determined states implemented by the commitment given by the primary signal *csd* and (2) maintain the determined state throughout development. That a positive feedback would generate stable determined states throughout development is demonstrated here by the finding of mosaic structures in *fem*-repressed females that have male or female characters, but not an intermediate phenotype. The mosaic intersexual phenotype is consistent with previous reports on a cell-autonomous sexual differentiation mechanism in the honeybee [42], but our data also provide evidence for the *fem* positive feedback loop as being the mechanism of stable determined cells.

We also explored how the female state is established and reset to the default male state in early embryogenesis. This is important, as male development is only induced in the absence of *fem* activity and as constant activity of the positive feedback loop would lock eggs into the female determined state. Our developmental profile of *fem* mRNA expression indicates that transcription of *fem* starts expression to sizable amounts of mRNA of the male type when blastoderm is formed (∼12 h after egg deposition). This finding implies that the sex determined state is set to the default male regulatory mode by the onset of *fem* transcription in early embryogenesis. The female pathway is induced in late blastoderm stage (∼25–35 h after egg deposition) when the female mRNAs in females are produced.

We also provide evidence that *csd* is required only to initiate sex-specific differentiation early in development. When we transiently repressed *fem* activity, we found male *fem* mRNAs under conditions of resumed mRNA production in late female larvae, suggesting that *csd* is no longer able to induce the production of female *fem* mRNAs. We conclude from this finding that *csd* can implement the female pathway only during a critical period of development. Primary signals from other organisms are also needed for only a critical developmental window when the pathway is established [22],[43]–[45]. In *Drosophila* [22] and possibly in the mouse [46] the sex determined state that is established by the primary signal is maintained later on in development by a different mechanism. In *Drosophila*, once the *Sxl* gene is activated in females by the primary signal (the X∶A ratio), the Sxl protein splices its own transcript into the productive female mode [34],[35]. This feedback splicing loop locks development into the female pathway as shown by mosaic intersexual analysis [36]. Our data also suggest that there are separate mechanisms for initiating and maintaining the sex determined state in honeybees. While honeybees and *Drosophila* utilize the same strategy in the control of the sex determined state, they employ different molecules.

### The *fem/tra* Gene Encodes Key Functions of Sex Determination that Are Ancestral among Holometabolous Insects

We have previously suggested, based on functional and some structural similarities, that the *fem* gene and the dipteran *tra* genes of *D. melanogaster* (*tra*) and of the medfly *Ceratitis* (*Cc-tra*) have a common evolutionary origin [19] irrespective of the great amino acid sequence differences between their proteins. The knowledge of *tra* functions in *Drosophila* [22] and *Ceratitis* [37],[38] and of *fem* studied here allows us to infer ancestral and derived functions within the *fem*/*tra* gene family (Figure 7A–7C). *Tra* and *Cc-tra* gene produce sex-specific transcripts and the encoding female Tra protein directs the splicing of *dsx* pre-mRNA into the female mode, implying that this pathway is conserved over the last 130 million years among this group of dipteran insects. The phylogenetic position of the honeybee is at the base of holometabolous insects [47] (including the Diptera [flies], Lepidoptera [moths, butterflies], and Coleoptera [beetles]) (Figure 7D), representing over 270 million years of evolution.

Most importantly, the sex-determining genes *fem*, *tra*, and *Cc-tra* are activated by processing the pre-mRNA into the productive female mode. In all the three species the female *tra*/*fem* mRNAs encode the active gene product, which directs the splicing into female *dsx* mRNAs. The male part of this hierarchy is also conserved: in the absence of the sex-specific signal the male mRNAs of the *tra/fem* and the *dsx* gene is produced, which is the default regulatory state. We conclude that despite great variety of sex determination mechanisms in insects [48]–[50] the processing of sex-specific information by the *tra/fem* gene in these insects is conserved. This implies that the sex determination pathways of holometabolous insects converge at the level of the *tra* gene family. The *fem* and the *Cc-tra* gene of *Ceratitis* are required for germ-cell differentiation, whereas in *Drosophila* the function of *tra* is restricted to the soma, suggesting that the control of the germ sexual identity is ancestral. Our comparison further indicates that the maintenance of the sexual fate at the level of the *tra* gene family is ancestral. Our mosaic analysis suggests that *fem* has an additional function in maintaining sex throughout development. We find direct molecular evidence for this in the form of a feedback splicing loop in which Fem proteins mediate splicing of *fem* pre-mRNA into the productive female mode. In *Drosophila* the female state is maintained via the positive feedback splicing loop of the *Sxl* gene, which operates as the next upstream regulator of the *tra* gene (Figure 7C). The repression analysis of *Cc-tra* and putative binding sites of Tra/Tra-2 proteins are indicators of a positive feedback splicing loop operating in *Ceratitis* at the level of the *Cc-tra* gene [37],[38]. This comparison indicates that the maintenance of sexual fate via a splicing loop of the *tra*/*fem* gene is most likely ancestral and that a self-splicing loop has been co-opted in *Drosophila* by the *Sxl* gene [51]. We infer that the maintenance of sexual fate in the sex determination pathway is a critical strategy in the development of a holometabolous insect. Taken together, the phylogenetic comparison suggests that the common ancestor of the *fem*/*tra* gene was employed in implementing and maintaining all visible aspects of sexual differentiation, while the upstream sex determination mechanism can vary. We conclude that the *fem*/*tra* gene is the ancestral key regulator of sex determination of holometabolous insects.

## Materials and Methods

### Bee Sources

Diploid female eggs were derived from the progenies of eight queens inseminated with semen from a single drone having a different sex allele than that of the queen. Diploid male eggs that are homozygous for *csd* were obtained from two queens that were derived from brother–sister crosses (inbred crosses), thus producing 50% female and 50% diploid male offspring. Haploid male eggs were collected from colonies that were headed by a virgin queen. These non-mated queens were laying unfertilized male eggs induced by repeated CO_2_ treatments.

### Characterization of Genes

Potential exons of the assembled SDL genomic sequence [19] were predicted by different methods that are included in the Gene Machine annotation software (http://genemachine.nhgri.nih.gov/) using the human or *Drosophila* model organism option and by different BLAST search strategies at NCBI (http://blast.ncbi.nlm.nih.gov/Blast.cgi). The first strand cDNA from mRNA was generated by reverse transcription with oligo dT primer (or random hexamers), and 5′ and 3′ ends of genes were identified by RACE experiments according to the protocol of the supplier (Ambion, Fermentas). Sequences of transcripts were obtained from high-fidelity PCR amplifications of embryonic cDNA and at least three independent clones. Potential homologies to genes and proteins in the database were identified by BLAST analysis using the low complexity filter option. Potential domains of proteins were identified by comparing deduced amino acid sequence with the PROSITE (http://www.expasy.org/prosite/) and the PFAM (http://pfam.sanger.ac.uk/) database.

### Functional Analysis

RNAi knockdowns were induced in early embryogenesis at the syncytial stage (0–4 h after egg deposition) in haploid and diploid males and females [16],[52]. dsRNAs were generated from cloned cDNAs of genes GB11211, GB13727, and GB30480. The mock dsRNA was generated from a DNA marker sequence [16]. The *fem* and *csd* siRNAs were synthesized (MWG BioTech) (Dataset S1, A). siRNAs were injected at a concentration of 50–100 pg per embryo. Sequences for the mock siRNAs (Dataset S1, B) were obtained by scrambling the nucleotide composition of *fem* and *csd* siRNA sequences. siRNAs were injected at a concentration of 50–100 pg per embryo. Individuals that were derived from inbred crosses were sexed according to the genotype at the *csd* locus [16]. Hatched larvae were reared in the incubator at 35°C and saturated humidity with food supply that consists of a mixture of glucose (3.6%), fructose (3.6%), and yeast extract (1%) dissolved in 52% royal jelly (weight per volume). Food supply was removed at the stage of 5^th^ instar larvae to allow pupation. Gonad tissue used for microscopic sections was fixed with 3% glutaraldehyde in 0.1 m sodium cacodylate buffer (SCB) pH 7.2 for 3 h at room temperature, washed with SCB, postfixed with 1% osmium tetroxide in SCB, dehydrated in a graded ethanol series, and embedded in epoxy resin [53]. Semithin sections (1 µm) were made with an ultramicrotome S (Leica, Bensheim, Germany) and stained with 1% toluidine blue. To quantify the mRNA levels with a BioRad Chromo4 cycler, total RNA was extracted from single 36-h-old embryos and transcribed in cDNA using random hexamer oligonucleotides. Aliquots of single stranded cDNA were amplified (Dataset S1, C), and real-time fluorimetric intensity of SYBR green was monitored. Each sample was run twice in triple replicates. ΔCts values were obtained by comparing cycle thresholds (Cts) to those of the reference gene, *elongation factor 1-alpha* (ΔCts = Cts_control_ − Cts_target_). *t* test statistics were carried out using the SPSS 15.0 software. Amplified fragments by *fem* RT-PCR (Dataset S1, D) in the repression experiments were composed of exons 3-6-7-8 (size ∼350 bp) and exons 3-4-5-6-7-8 (size ∼1.6 kb) corresponding to the female and male transcripts, respectively. Amplified fragments in the *Am-dsx* RT-PCR experiments (Dataset S1, E) were composed of exons 3-4-5-6-7 (size ∼1.4 kb) and exons 3-4-6-7 (size ∼500 bp) corresponding to the female and male transcripts, respectively. Amplified fragments (Dataset S1, F) in the *fem* developmental profile analysis were composed of exons 3-6-7-8 (size ∼350 bp) and exon 3 (size ∼400 bp) representing the female and male transcripts, respectively. The cDNAs in the profile analysis were quantified in a NanoDrop ND-1000 spectral photometer. Differences in the amount of cDNAs were adjusted prior to PCR amplifications. mRNAs (*fem^csd-UTR^* mRNA) encoding Fem proteins were generated by inserting 5′ and 3′ UTRs at the corresponding 5′ and 3′ end of the *fem* ORF of clone fem S2-38 [18]. We first inserted the 5′ *csd* UTR together with a translational start codon and a Myc tag encoding sequence between the ApaI und NcoI restriction sites of the pGEMT vector (Promega). The *csd* 3′ UTR was inserted by utilizing SpeI und PstI restriction sites. The *fem* ORF was ligated into NcoI and SpeI resulting in the plasmid pfem^csd-UTR^. The mRNA (*fem^csd-UTR^* mRNA) was generated using the RiboMax T7 RNA polymerase kit (Promega) in which the 5′cap structure (Ambion) was added during RNA synthesis in order to produce 5′ capped transcripts. We polyadenylated the 3′ termini of the in vitro transcribed RNA by adding ATP and Yeast polyadenylation polymerase (USB), which we terminated by following standard phenol/chloroform extraction protocol. We injected 0.08 pg of *fem^csd-UTR^* mRNA into 0–3-h-old male embryos. Amplified fragments (Dataset S1, G) of endogenous *fem* were composed of exons 2-3-6 (size ∼560 bp) and exons 3 (size ∼400 bp) corresponding to the female and male transcripts, respectively.

## Supporting Information

Dataset S1 siRNA and oligonucleotide primer sequences.
(0.03 MB DOC)

## Abbreviations

*Cc-tra*: *Ceratitis capitata* at the level of the sex-determining tra
*csd*: *complementary sex determiner*
Cts: cycle thresholds
*dsx*: *doublesex*
*fem*: *feminizer*
PCR: Polymerase Chain Reaction
RT-PCR: Reverse Transcriptase Polymerase Chain Reaction
SCB: sodium cacodylate buffer
SDL: Sex Determination Locus
*Sxl*: *Sex-lethal*
*tra*: *transformer*
