# Supplementary material for: Sex Determination in Honeybees: Two Separate Mechanisms Induce and Maintain the Female Pathway
Source: PLoS Biol. 2009 Oct 20;7(10):e1000222. doi: 10.1371/journal.pbio.1000222 (PMC2758576; doi:10.1371/journal.pbio.1000222)
Supplement: Dataset S1 — siRNA and oligonucleotide primer sequences. (0.03 MB DOC) [file pbio.1000222.s001.doc]

**Dataset S1**

**A) siRNA sequences for RNAi-induced knockdowns:**

*fem*:

fem_siRNA_1: GAUUCAAGACAUGAAGACA

fem_siRNA_2: AUGGUUAGACCUUGGGUUC

*csd*:

csd_siRNA_1: CGUAUUCGUGAAAUAGAAA

csd_siRNA_2: CAAUGGGACCUUGGAUUUC

**B) Mock siRNA sequence:**

sc1_siRNA: UACAUAGAAGAGAGAUCAC

sc2_siRNA: GUCAUCCGAGGUGUAUUCA

**C) Oligonucleotide primer sequences designed for real-time PCR amplifications:**

*GB11211* transcript:

GB11211_64_ fw: tgctgcgagcaattcgaatag

GB11211_65_rv: GGGCCTGTCCAATGTCTAAC

*GB13727* transcript:

GB13727_70_fw: CATGCTTTCTGCATTGAATCTAC

GB13727_71_rv: GACCAATCACAGCCATTAGTG

*fem* transcript:

Tra_863_fw: AACGTACATCATCCTGTCATTC

Tra_982_rv: GCAATTGGTCATATCGTCTATC

*csd* transcript:

csd_121_CR_fw: GAAGCRTGGTTGATACAACAAG

csd_260_GR_rv: YATTACTTCTATCACGACTATCTG

(with Y designating a pyrimidine)

**D) Oligonucleotide primer sequences for PCR amplifications designed to amplify the sex-specific splice variants of *fem*** **in the repression experiments:**

Female and male *fem* transcript:

TypeII-W2: CAACATCTGATGAACTTAAACGG

Race_typeII_rv2: CTATTTCTGTCTTCATGTCTTGAA

**E) Oligonucleotide primer sequences for PCR amplifications designed to amplify the sex-specific splice variants of *Am-dsx* in the repression experiments:**

Female and male *Am-dsx* transcript:

dsx6_fw: TGGTCACCCATTTGCCACAGAC

M550_rv: TCCTCTTTGGATTTGACCTGTTCT

**F) Oligonucleotide primer sequences for PCR amplifications designed to amplify the sex-specific splice variants of *fem* in the developmental profile analysis:**

Female *fem* transcript:

TypeII-W2: CAACATCTGATGAACTTAAACGG

Race_typeII_rv2: CTATTTCTGTCTTCATGTCTTGAA

Male *fem* transcript:

TypeII-W2: CAACATCTGATGAACTTAAACGG

tra-likeM_781_rv: GGATTCAAATCATCTGAAGTTAC

**G) Oligonucleotide primer sequences for PCR amplifications designed to amplify the sex-specific splice variants of *fem* in the *fem^csd-UTR^* mRNA injection experiments:**

Female *fem* transcript:

FemUTR_fw: TTTGCAGATTAAATTTCATAAATATATATA

FemEx6_rv: TTCTCTTTCAAATATAGGCTTAGATCCTTCT

Male *fem* transcript:

TypeII-W2: CAACATCTGATGAACTTAAACGG

tra-likeM_781_rv: GGATTCAAATCATCTGAAGTTAC
